# The Role of Colony Size on Tunnel Branching Morphogenesis in Ant Nests

**DOI:** 10.1371/journal.pone.0109436

**Published:** 2014-10-15

**Authors:** Jacques Gautrais, Camille Buhl, Sergi Valverde, Pascale Kuntz, Guy Theraulaz

**Affiliations:** 1 Centre de Recherches sur la Cognition Animale, Université de Toulouse, Toulouse, France; 2 Centre de Recherches sur la Cognition Animale, Centre National de la Recherche Scientifique UMR5169, Toulouse, France; 3 School of Agriculture, Food and Wine, The University of Adelaide, Adelaide, Australia; 4 Complex Systems Laboratory (ICREA-UPF), Barcelona, Spain; 5 Institut de Biologia Evolutiva (CSIC-UPF), Barcelona, Spain; 6 Laboratoire d'Informatique de Nantes-Atlantique, Polytech'Nantes, Nantes, France; University of Freiburg, Germany

## Abstract

Many ant species excavate nests that are made up of chambers and interconnecting tunnels. There is a general trend of an increase in nest complexity with increasing population size. This complexity reflects a higher ramification and anastomosis of tunnels that can be estimated by the meshedness coefficient of the tunnelling networks. It has long been observed that meshedness increases with colony size within and across species, but no explanation has been provided so far. Since colony size is a strong factor controlling collective digging, a high value of the meshedness could simply be a side effect of a larger number of workers. To test this hypothesis, we study the digging dynamics in different group size of ants *Messor sancta*. We build a model of collective digging that is calibrated from the experimental data. Model's predictions successfully reproduce the topological properties of tunnelling networks observed in experiments, including the increase of the meshedness with group size. We then use the model to investigate situations in which collective digging progresses outward from a centre corresponding to the way tunnelling behaviour occurs in field conditions. Our model predicts that, when all other parameters are kept constant, an increase of the number of workers leads to a higher value of the meshedness and a transition from tree-like structures to highly meshed networks. Therefore we conclude that colony size is a key factor determining tunnelling network complexity in ant colonies.

## Introduction

Most species of ants excavate their nest and form a subterranean network of tunnels that connect several chambers [1], [2], [3], [4], [5], [6], [7]. These chambers are ellipsoidal cavities used to raise brood, store seeds, grow fungus or accumulate litter and corpses. Sometimes they may also be used as resting places in which dense aggregations of workers can be found. The tunnels ensure the connection of the underground parts of the nest to the soil surface. Previous field studies [8], [9], [10] and controlled laboratory experiments [6], [11], [12], [13], [14] have shown that nest size was proportional to the size of a colony in several species of ants. In addition to the adjustment of nest volume, the variations of ant nest architecture with colony size result in a different number of chambers and tunnels. The simplest nests are made up of a few chambers stacked along a single vertical tunnel, whereas dense networks of tunnels with a large number of interconnected chambers are found in the most complex nests. So far no correlation has been established between the observed variations in the complexity of nest architecture and phylogeny [15], [16], [17]. In most ant genera, nests are subterranean excavated networks, but within each particular genus, there exists a large variability of nest size and complexity. As a general trend, however, nest complexity increases with colony size [8], [13], [18], [19] (see Table 1).

**Table 1:** The relationship between meshedness and colony size in ants.

Species	Pop. Size	Architecture	References
*Ectatomma opaciventre*	9–61	Single vertical tunnel	Antoniolli-Jr 1997
*Amblyopone pluto*	18–35	Single vertical tunnel	Lévieux 1976
*Aphaenogaster sp.*	200	Single vertical tunnel	Tschinkel 2003
*Odontomachus brunneus*	300	Single vertical tunnel	Tschinkel 2003
*Myrmecia dispar*	368	Stacked horizontal tunnels connecting one vertical shaft	Gray 1971
*Mycetophylax simplex*	68–611	Stacked chambers connecting one vertical tunnel	Diehl-Fleig 2007
*Conomyrma sp.*	1000	Stacked chambers connecting one vertical tunnel	Tschinkel 2003
*Messor arenarius*	∼1500	Numerous inter-connected chambers.	Délye 1971
*Myrmecia brevinoda*	2576	High meshedness under the mound	Higashi 1990
*Formica pallidifulva*	2946	High meshedness decreasing with the depth (“top-heavy”)	Mikheyev 2004
*Pheidole Morrisi*	6000	Top-heavy	Williams 1988; Tschinkel 2003
*Pogonomyrmex badius*	8000	High meshedness at shallow depths and top-heavy	Tschinkel 1999a
*Prenolepis imparis*	11000	High meshedness at shallow depths and one descending tunnel	Tschinkel 1987
*Formica exsectoides*	238,000	Very high meshedness	Bristow 1992
*Solenopsis invicta*	250,000–500,000	Very high meshedness in a compact nest. Numerous tunnels and chambers	Tschinkel 2003

Quantitative descriptions of nests dug in natural conditions are difficult to obtain. Structural features of a hypogeous nest can be quantified with plaster or aluminium casts at a given moment of colony life [20], [21], [22]. However these methods cannot be used to follow the development of the nest structure over time. Hence we investigated the dynamics of collective digging in controlled laboratory conditions, allowing groups of ants to dig into horizontal moistened and homogeneous disks made of sand. We previously characterized the topological structure of the networks built by three group sizes (50, 100 and 200 workers) [11]. In a subsequent paper, we analyzed the tunneling dynamics using the dataset obtained with 200 workers and formulated a model for the network growth in which the tunnels were the basic units, focusing on their birth, growth and the intersection events between tunnels [23]. We then suggested that a next step should be a sensitivity analysis of the network topology to the model parameters. However, preliminary numerical explorations showed that the number of workers might be worth further investigation. Here to investigate the impact of this parameter on the resulting network structure, we use the dataset obtained with the three group sizes and we introduce a new model that incorporates explicitly the ants digging activity level. We test the predictive power of the model for the networks meshedness with 50, 100 and 200 ants respectively. We then explore the predictions of the model over a large range of colony sizes in which collective digging progresses outward from a centre. In these conditions, the model predicts that the meshedness rapidly increases with the number of workers and then reaches a plateau at a critical size. We conclude that an increase of the number of workers is a sufficient condition to induce a transition from tree-like to highly branched and meshed structures.

## Methods

### Ethics statement

Colonies of ants Messor sancta were collected in southwestern France (Narbonne), on a private property with the permission of the owner. Messor sancta is not a protected nor endangered species. Our experiments complied with the laws and ethical recommendations currently in effect in France where the experiments were performed.

### Experimental setup and data collection

**Figure 1:**
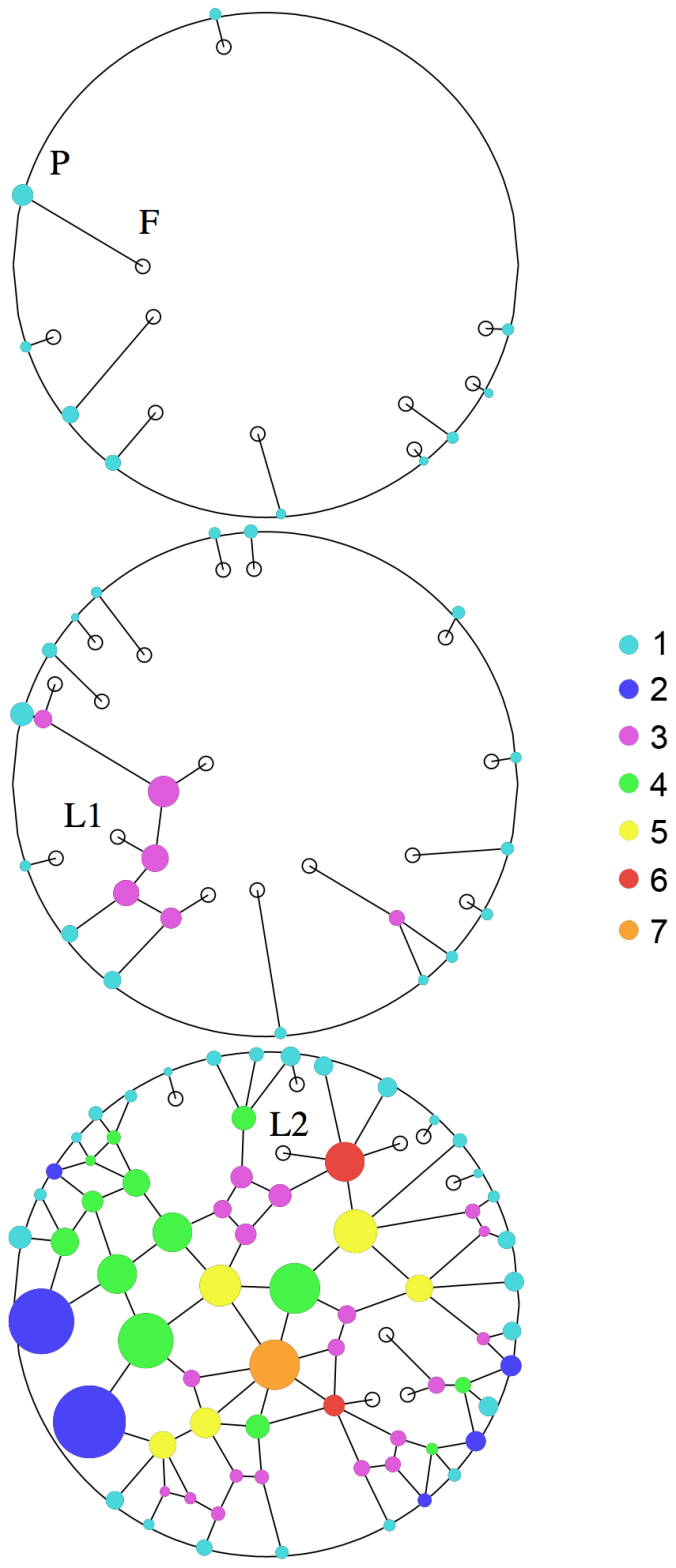
Typical evolution of a tunnelling network dug by A(0) = 200 ants, at 5, 9 and 72 hours after T0. The outer circle represents the periphery of the sand disk (radius 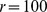 mm). Plain disks represent chambers, and lines represent tunnels. Open disks denote active fronts (F), progressing from a peripheral node (P), from an existing gallery (L1) or from an existing node (L2).

Let us briefly recall the main features of the experiments described in [11]. We recorded and quantified the digging activity of groups of *Messor sancta* ants in a two dimensional and uniform environment. The general experimental set-up consisted of a disk of sand (radius 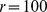 mm, height 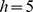 mm). We used fine yellow homogeneous sand which was poured into a mould and humidified by spraying with water (25 ml). The mould was then removed and the disk of sand covered by a glass plate (25 cm×25 cm). To prevent ants from escaping, an arena (diameter 50 cm) with a wall coated with Fluon GP2s was placed around the disk. With this set-up lit 24 hours a day, ants' digging activity was strongly stimulated and workers dug 2D tunnel networks in the sand within three days (fig. 1, movie S1). We recorded the digging dynamics with three different group sizes of workers: 50 (n = 5), 100 (n = 5) and 200 (n = 18), for three days (T = 4320 minutes) with a high-resolution digital camera (SONY DCR-VX1000E). Snapshots of the evolution of the network were taken every 20 minutes with a total of 217 snapshots per experiment. The width and position of tunnels and nodes were recorded. We also took into consideration the way nodes have been created either initiated from the periphery of the sand disk, from an existing tunnel or from the collision between two existing tunnels. These data are available as Data S1.

### Model of collective digging

In the model, ants dig into a 2-D plane sand disk of radius 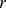, starting from the periphery and progressing toward the centre. As time goes on, existing tunnels extend in a straight line with active fronts at their end. New tunnels are dug either from the border of the sand disk or from the sides of an existing tunnel. In the latter case, this results in a branching structure. The width of tunnels is set to a constant value 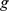. When an active front collides with another tunnel, a node is created at the collision point, with a circular surface of diameter 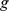: 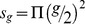. When it collides with a node, the surface of the existing node is increased by 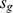 (following [23], see fig. 6b therein).

The rate at which the network structure changes ( ∼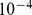 cm/s [23]) is much slower than the rate at which the ants move inside the network ( ∼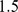 cm/s [24]). Hence, the diffusion of ants within the network occurs at a much faster time scale than the growth rate of the network. As a consequence, there is no benefit to model each ant's individual position and trajectories explicitly because ants are statistically likely to make digging decisions on a large sampling of the whole network. Instead, we can derive analytical descriptions for the dynamics of the network components (i.e. the number of nodes and the length of tunnels) by integrating ant activity over time, with no explicit rendering of individual digging activity. Ants are then considered as a global work force uniformly distributed all over the network at any given time. This abstracted representation of the spatial distribution of ants is also relevant because there is no interaction between the individuals in the model (e.g. no trail following).

Initially, the entire population of ants is active and set to 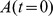 individuals. This pool decreases with time at a constant rate 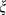 (ant.min^−1^) so that the number of digging ants at time t is:
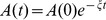
(1)

A small amount of time 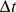 is required for an ant to dig a small length of gallery 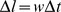 (with 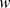 in mm.ant^−1^. min^−1^). At time t, the resulting dug length is then:
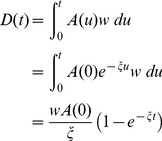
(2)

The total work force at time t, 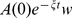, is equally shared on each progressing front. Ants not only increase the length of existing tunnels; they may also start new tunnels. First, they may start a new digging front on the border of the sand disk at a constant rate 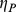 (expressed in ant^−1^.min^−1^.mm^−1^). This creates a peripheral node whose size is set to 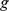. The new digging sites can only be created along the unaltered portion 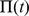 of the periphery (i.e. the perimeter which is not already occupied by nodes), and this portion decreases as the number of peripheral nodes increases. The direction of a new tunnel is perpendicular to the tangent of the sand disk with a Gaussian noise of standard deviation 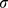. At time t, the resulting number of peripheral nodes is:
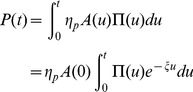
(3)

Ants may also start to dig a new internal front at a constant rate 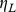 thus creating a new branch in the network. This new tunnel can be dug either along the wall of an existing tunnel with a probability 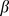, or from an existing node with a probability 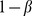. In the first case, the digging site is randomly and uniformly chosen along the existing tunnels, a lateral node is created at that place, and the new lateral front progresses at right angle to the originating tunnel, with a Gaussian noise of standard deviation 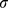. In the second case, the node is uniformly chosen among the internal nodes, and the orientation of the new tunnel is uniformly distributed.

Since lateral fronts can only emerge along the walls of existing tunnels whose total length 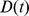 increases with time, 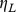 is expressed in ant^−1^.min^−1^.mm^−1^. At time t, the resulting number of lateral nodes is then:
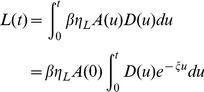
(4)

The parameters of the model are: the number of ants 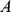, the radius of the sand disk 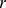, the total time 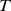, the geometrical parameters 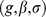 and the behavioural parameters 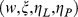 (Table 2).

**Table 2:** Parameters of the model.

Parameter	Symbol	Units
Number of ants	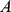	ants
Radius of the sand disk	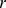	mm
Total duration of the digging activity	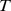	_min_
Noise in the orientation of new tunnels	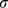	degree
Probability that a new internal gallery starts from a node rather than from the edge of a gallery	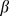	
Tunnel width	_g_	mm
Individual digging rate	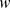	mm.ant^−1^. min^−1^
Inactivation rate	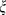	ant.min^−1^
Initiation rate of new tunnels from the periphery	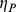	ant^−1^.min^−1^.mm^−1^
Initiation rate of new tunnels from existing tunnels or nodes	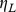	ant^−1^.min^−1^.mm^−1^

### Estimation of model parameters

**Figure 2:**
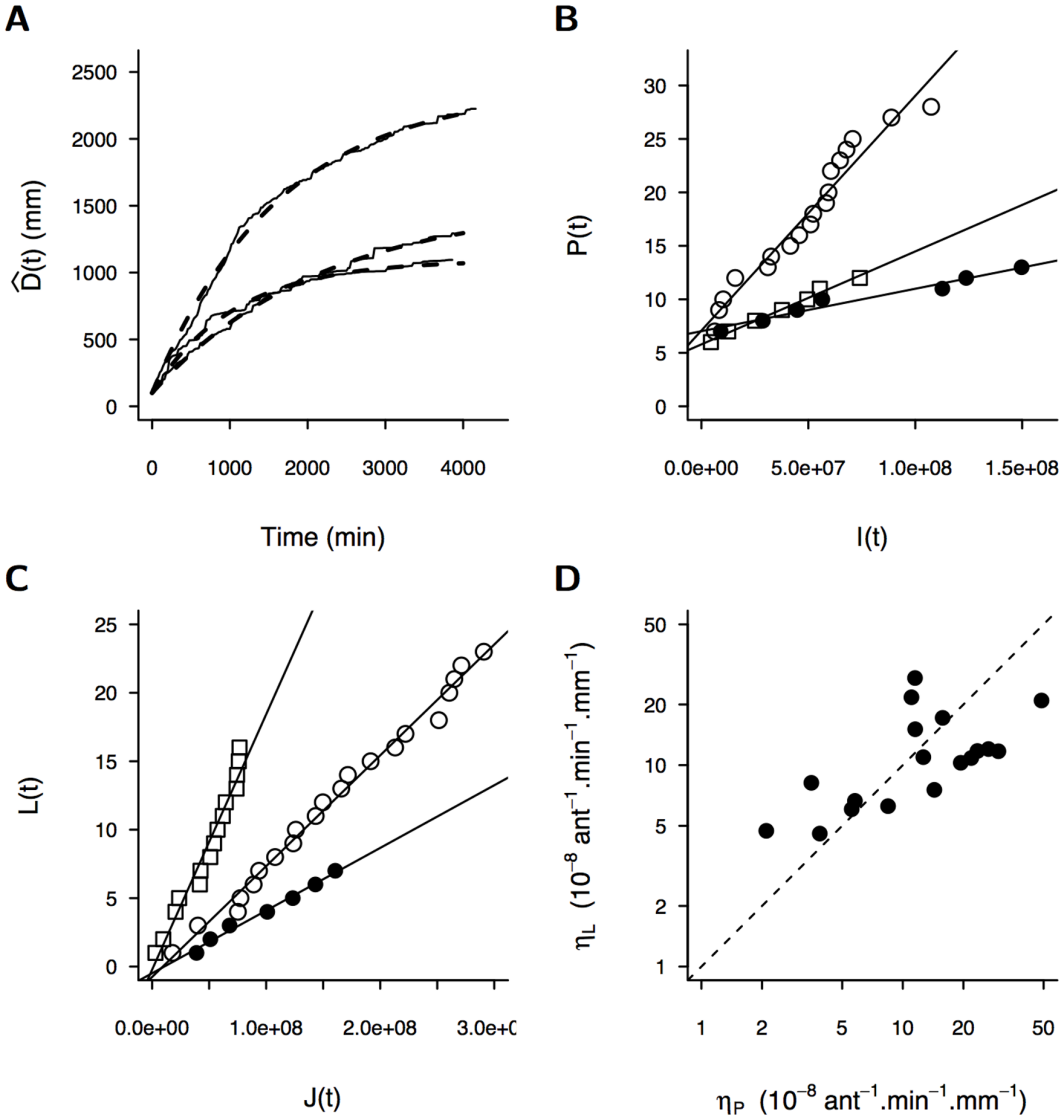
Quantification of the parameters of the digging dynamics from experimental data with A(0) = 200 workers. (a) Ant activity parameters (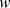 and 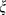) are retrieved from the evolution of the dug length 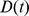: three typical experimental cases are shown as solid lines and their corresponding predictions by the model as dashed lines (see also fig. S1 for all experimental results). (b, c) The rates of initiation of new peripheral nodes 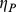 and of new lateral nodes 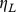 are retrieved respectively from (b) the regression slope of the number of peripheral nodes 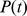 as a function of 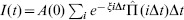 and (c) the number of lateral nodes 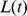 as a function of 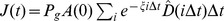 (each symbols corresponds to each of the three experiments shown in (a)). Regressions for all cases can be found in fig. S2 and S3. (d) The rates of initiation of new lateral 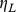 and peripheral 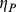 nodes converge to a common value (one symbol per experiment, dashed line: 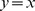).

**Figure 3:**
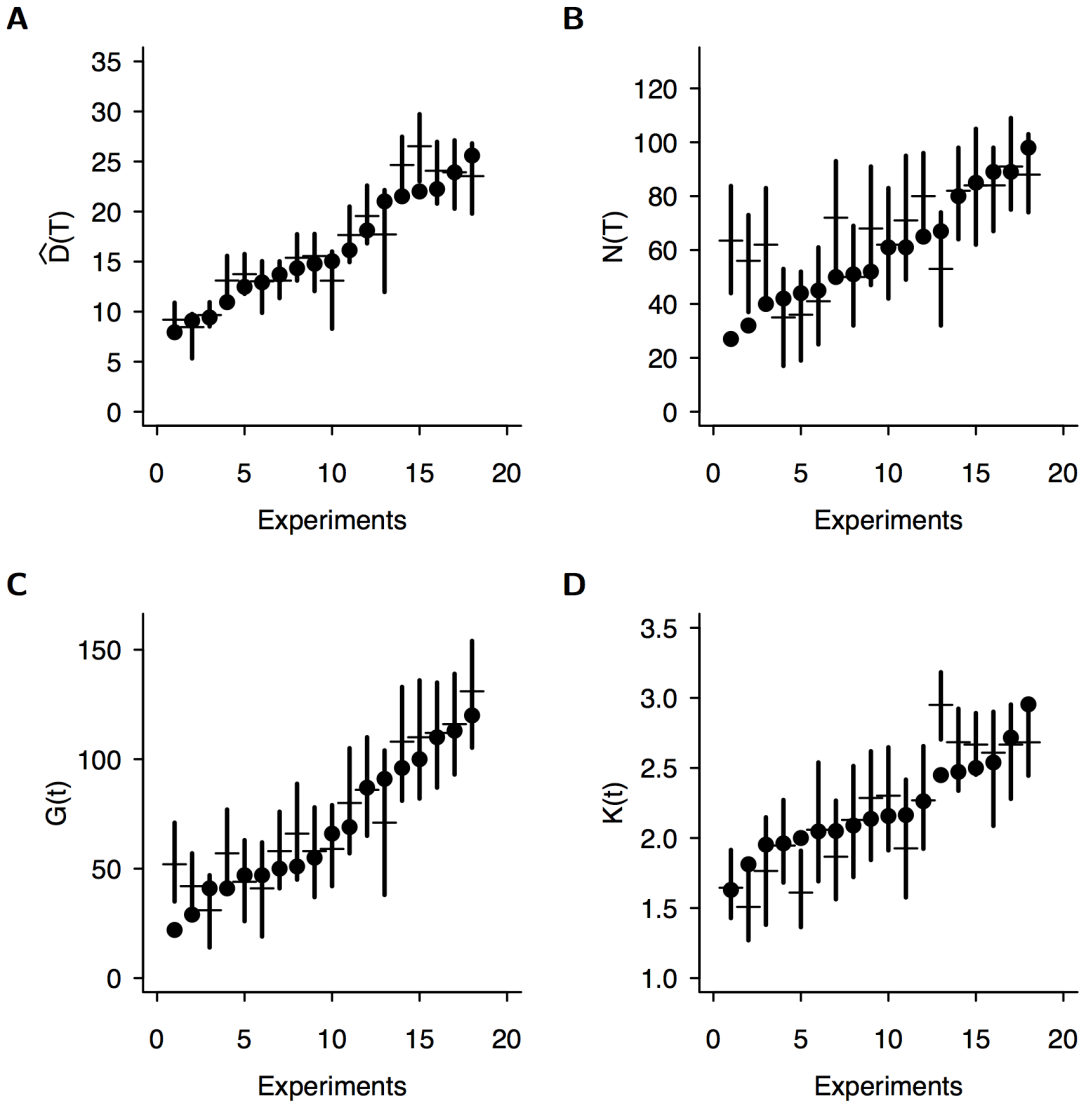
The structural properties of the observed networks at the end of experiments after three days (one symbol per experiment) are well predicted by the model for (a) the length 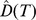, (b) the number of nodes 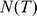, (c) the number of tunnels 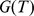 and (d) the mean degree 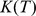. In each plot, vertical bars indicate 95% confidence interval predicted by the model and were numerically estimated using simulations (N = 2000 simulations per experiment using the corresponding set of behavioural parameters). Horizontal bars indicate the predicted median. Note that experiments have been ordered by increasing ordinate values.

A total of 18 experiments, all performed in the same conditions, with A(0) = 200 ants were used to estimate the model parameters. To compensate for delays in the initial phase (ants do not start digging into the sand disk with the same latency after their introduction in the setup), the initial time 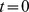 of each experiment was set when 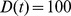 mm, which represents about 5% of the final length. The geometrical parameters of galleries and nodes appeared to be rather homogeneous across the networks and time, so they were estimated by averaging the values got over the dataset. In contrast, the evolution of the topology (new peripheral nodes, branching or collision, elongation) appeared to obey a non-linear stochastic process, responsible to the high variability observed in the growth dynamics of the networks (Fig 2a, S1). Since the stochastic events involved in the growth dynamics depend at any time on the state of the network, each experiment has a particular history, meaning that the development of each particular network is following one stochastic path within the space of all possible networks. Therefore in first step, we estimated the behavioural parameters 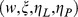 separately for each experiment (one set of parameters per experiment). To check that the procedure for estimating these parameters was efficient, we then tested the model predictions for each case (fig. 3). Then, in a second step, we tested the predictive power of the model despite this variability.

#### Parameters of network components

The estimation of the standard deviation of the orientation of new tunnels, the mean width of tunnels and the probability of branching from tunnels or nodes, were computed from all experimental data. We found respectively 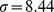 deg, 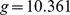 mm and 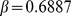 [23].

#### Ants activity

We used the temporal evolution of the networks to estimate the behavioural parameters. Ant activity parameters, 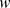 and 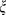, were estimated from the length of tunnels dug by ants 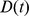 over the 217 observed snapshots. The evolution of 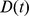 with time is not strictly monotonous, because two nodes close to each other can merge when they are both hit by a progressing front. 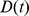 was thus recovered by cumulating the differences of the observed length dug between successive snapshots when positive, yielding 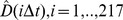.

In a first step, we obtained a rough estimation of 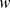 from the initial slope of 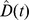 during the first 1000 minutes by:
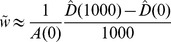
(5)and 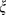 was estimated at the end of the experiment by numerically solving for the root of the implicit equation:
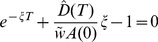
(6)

In a second step, these approximate values were refined jointly by a Nelder-Mead minimization of the prediction error, computed as the sum of the squared differences between the 217 observed and predicted values (Least-square estimates).

Over the 18 experiments, we found 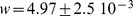 mm.ant^−1^.min^−1^ (mean±SD) and 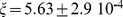 ant.min^−1^ (mean±SD). The parameter values for each experiment yield accurate predictions of the growth dynamics and the length of tunnels (fig. 2a and S1). This agreement between the model and observations strongly supports the hypothesis that ants' activity decays with time.

#### Peripheral nodes formation rate

In the model we assume that the number of peripheral nodes 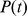 is given by (3), and depends on the integral over time of the length of unaltered periphery 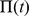. 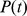 can be written as follows:
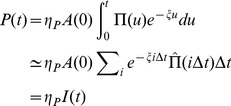
(7)with 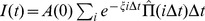 where 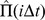 corresponds to the unaltered portion of the periphery, that is the length of disk perimeter minus the length occupied by existing peripheral nodes at times 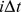 with i = 1,…217. The rate 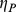 was then estimated with a linear regression of 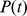 on 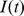. Over the 18 experiments, we found 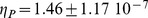 ant^−1^.min^−1^.mm^−1^ (mean±SD). The compelling linearity of the correlation for each experiment (fig. 2b and S2, 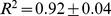) strongly supports the assumption that 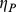 remains constant over time.

#### Lateral nodes formation rate

In the model we assume that the number of lateral nodes 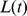 is given by (4), and depends on the cumulated length of the network over time. 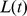 can be written as follows:
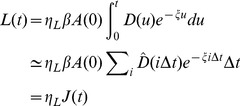
(8)with 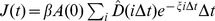 and 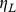 was estimated with a linear regression of 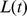 on 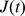. We found 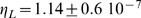 ant^−1^.min^−1^.mm^−1^ (mean±SD). Here again, the high linearity of the correlation for each experiment (fig. 2c and S3, 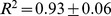) strongly supports the assumption that the formation rate of lateral nodes remains constant over time.

The similarity, despite independent quantifications, of lateral and peripheral nodes formation rates 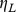 and 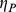 (fig. 2d) suggests that ants do not exhibit different behaviours when they start to dig a new tunnel from the periphery or from an existing tunnel inside the sand disk. This result also supports our choice to quantify the ants' propensity to dig a new tunnel with a probability per ant per minute as a function of the length of available walls (i.e. the sum of the unaffected part of the periphery for 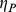 and the length of tunnels for 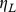).

### Quantification of networks properties

The result of the digging dynamics is a 2-D network that grows over time. We characterized this network with five graph-related measures: (i) the total length 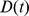, (ii) the total number of nodes 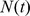, (iii) the number of edges (i.e. the subsections of tunnels connecting two nodes) 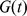, (iv) the average node degree 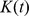 (i.e. the average number of tunnels per node) and (v) the meshedness coefficient 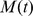.

The meshedness coefficient 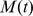 was introduced by Buhl et al. [25] to measure the structure of cycles in planar graphs and it is calculated the following way: 
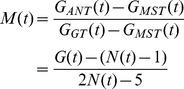
(9)

It is the number of tunnels 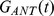 in the observed network, scaled within two extreme values that correspond respectively to a minimal spanning tree (MST, for which 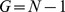) and a triangulated graph (GT, for which 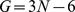, following Euler's polyhedra formula). 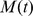 is a structural property of the network which indicates the density of edges regardless of the number of nodes. 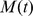 varies from 0 in tree-like networks to 1 in triangulated networks (maximally connected planar graph). The predictions of the model were compared with the networks quantities N, G, K and M estimated from the experiments.

## Results

### Structural properties of networks at the end of experiments

When the experiments ended, the average length of the networks was 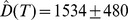 mm (corresponding to an average length of tunnel dug per ant 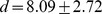 mm.ant^−1^), the average number of tunnels was 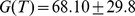, the average number of nodes was 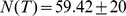, and the average node degree was 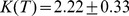.

Since there was a strong variability among experiments, we tested the correctness of the procedure used to estimate the parameters for each experiment separately. 2000 numerical simulations were performed with the set of parameters estimated for each experiment, for a total time of T = 4320 minutes corresponding to three days, with a time step 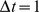 min.

We computed the predicted confidence intervals for the total length of the network 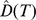, the number of nodes 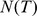, the number of edges 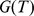 and the average node degree 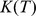. In almost all cases (8 outliers out of 72 measures), we found a very good agreement between the model's predictions and the experimental results (fig. 3). Of course, the length of the total network 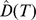 simply results from the ant activity parameters (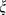 and 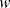) that have been fitted to get the expected results. However the average node degree, and the number of nodes and edges result from a complex sequence of growth, collisions and merging. At any time 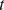, the total number of nodes 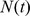 not only includes peripheral 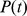 and lateral 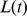 nodes, but also those that are created by the collisions of active fronts with existing tunnels or with the periphery. The average node degree 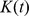 results from new digging fronts starting from existing nodes, but also from digging fronts that collide with existing nodes or tunnels. The number of edges 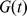 results from new digging fronts that strongly depend on the evolution of the number of peripheral nodes (through the unaffected space in periphery), the length of existing tunnels (through the branching rate) and the number of internal nodes (through the initiation of new fronts departing from nodes). Therefore it would have been very unlikely to get such a good fit across all experiments if the model had not described correctly the underlying dynamics [26]. This good agreement is further supported by the fact that the differences among predicted characteristics were significant across all experiments due to their variability (in fig 3, the 95% confidence intervals do not overlap).

### Effect of colony size

**Figure 4:**
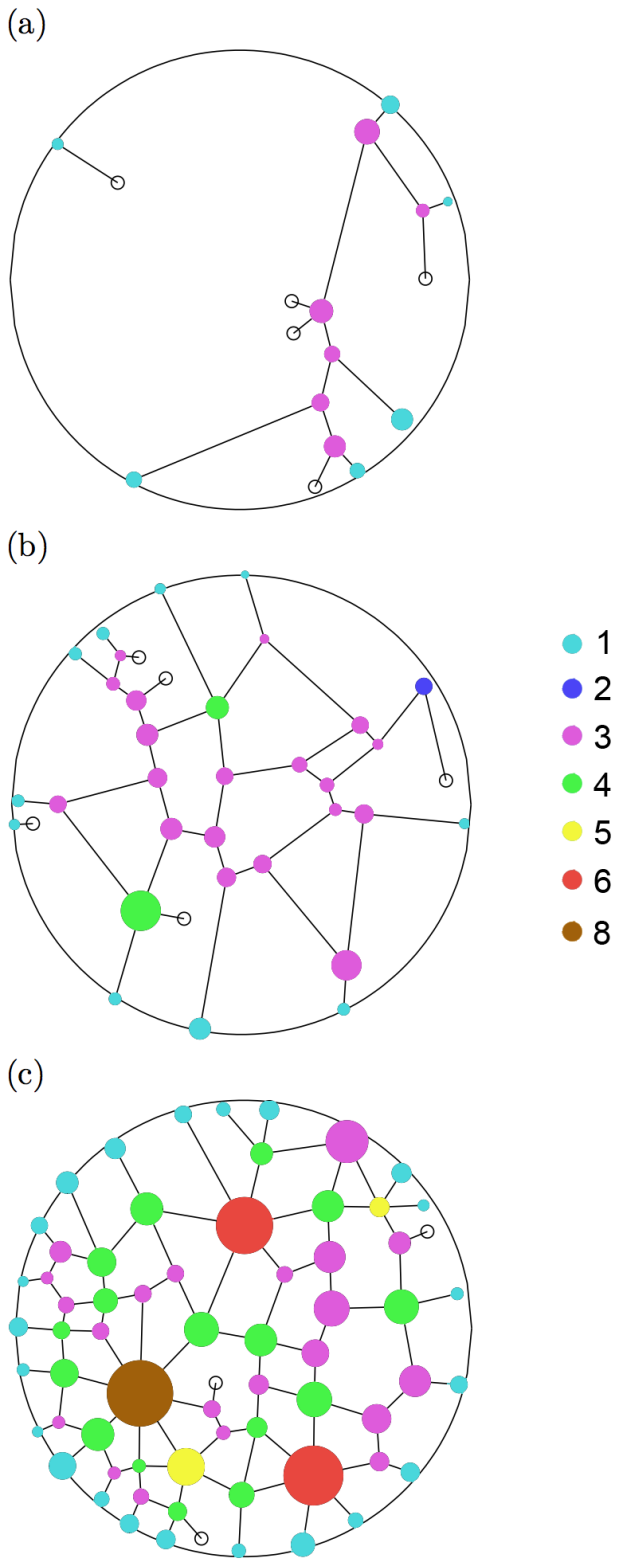
Examples of experimental networks dug by the ants in 72 hours depending on the number of workers (a) 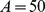, (b) 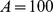, (c) 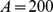. The corresponding values of the meshedness 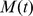 are respectively 0.0, 0.092 and 0.194.

**Figure 5:**
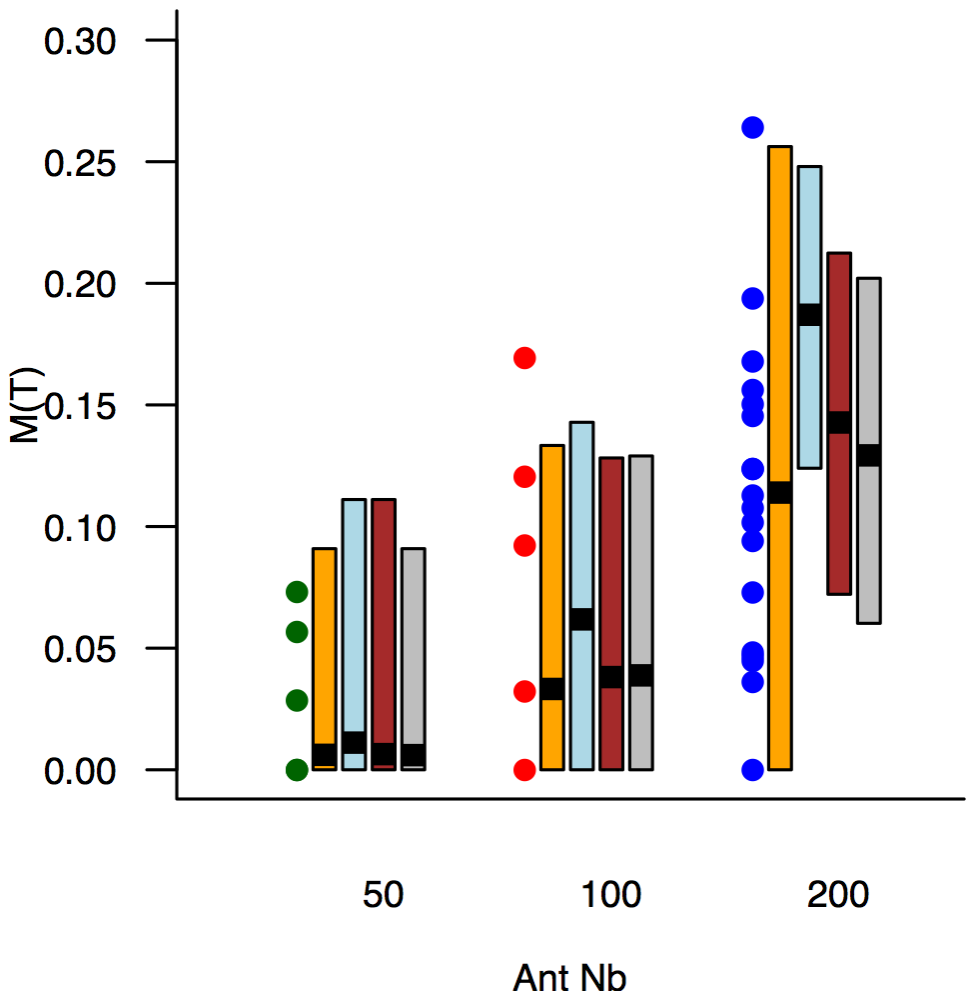
The impact of colony size on the final topology is shown by the meshedness 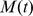 as a function of the number of workers 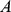. Symbols: experimental values for the three group sizes (green: A = 50, red: A = 100, blue: A = 200, same colours as in Fig. S4). Orange boxes: means and 95% CI predicted by the model using the complete collection of parameters sets. Blue boxes: means and 95% CI predicted by the model using the set of averaged parameters. Brown boxes: means and 95% CI predicted by the model using averaged branching rates, and activity parameters corresponding to the average activity. Gray boxes: 95% CI predicted by the simplified model using the same parameters.

Remarkably, when we increased the number of workers in the experiments (A = 50, 100, 200), we observed that the meshedness of the resulting tunnel networks increases (see some examples of networks in fig. 4, and data points in fig. 5). As a consequence, the average path length between any two nodes in the network remained close to a minimum value.

To investigate the impact of colony size, we had to deal with the observed experimental variability of the networks. Since the digging process is non-linear, there is no *a priori* reason that the model's predictions using average parameters should yield the average results. Indeed, if we call 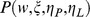 the process involved in the networks formation, it generates a non-linear dynamics that does not obey the superposition principle so that:
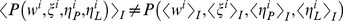
(10)where 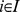 denotes the different sets of parameters, and 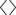 denotes the average over the sets. As a first check, we used the complete collection of parameters sets (corresponding to the left hand side term in (10)), performing 1000 simulations for each set and each group size, and combining the outputs. The results show an increase of the meshedness when group size increases together with an increase of variability for each group size (fig. 5, orange bars). As a matter of fact, we found that the variability of the meshedness results from the growth dynamics of the networks driven by parameters 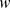 and 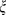, which is itself highly variable. As it can be seen on figure S4-A (orange curves corresponding to each couple 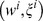), the total length of tunnels dug by groups of 200 ants can vary by a factor 3. The longer networks display a higher meshedness as expected (fig. S4-B).

In a second check, we used the set of average parameters (corresponding to right hand size term in (10)), performing 1000 simulations for each group size. The results predict on average longer networks and higher meshedness than observed in the experiments (fig. 5, blue bars). This can be explained by the fact that the dynamics of network growth using average values 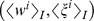 (fig. S4-A, blue curve) corresponds to a higher activity for a longer time than the average dynamics (fig. S4-A black curve). It is well known that averaging exponential decays over a set of different time constants will not yield the same results as an exponential decay corresponding to the average of the time constants.

In order to take into account this non-linear effect, we then performed a third check, using 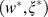 corresponding to the average activity dynamics 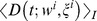 (fig. S4-A brown curve), and using the average peripheral and lateral branching rates. The results correspond to most of the observed data, except that the predictions for A = 200 do not cover the four shortest experimental networks.

Overall, the model confirms that group size has a strong impact on the meshedness (fig. S4-B). Beyond the variability of the activity profiles over time, the model captures the structural feature of the dynamics: keeping the behavioural parameters unchanged, the observed increase of the meshedness is driven by group size.

Finally, we tested a simplified version of the model in which the node surface does not increase after a collision. Simulations of this simplified model using 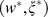 provide similar results namely an increase of the meshedness when the number of workers increases (fig. 5, gray bars). This is an indication that the geometrical details resulting from the collisions between a digging front and an existing node are not essential ingredients to account for the networks topology resulting from the digging process.

### Model predictions in a space with no boundaries

Since in the experiments the total length of the network increases with the number of workers, the observed increase of the meshedness could simply result from the spatial constraints imposed by the geometry of the sand disk. A network extending within a fixed area is likely to have more nodes with a higher connectedness (e.g. a higher mean degree). Even if the meshedness defined above is independent of the number of nodes, the centripetal nature of digging could have simply promoted a richer connectivity. To test the model predictions without this constraint, we changed the initial conditions, allowing the ants to dig a 2D-network extending outwards from a single starting point.

We measured the predicted meshedness of tunnel networks when the number of ants was varying from A = 1 to 10,000, with the simplified version of the model keeping the node surface to a constant value. In order to assess specifically the effect of a variation of the number of workers on the resulting network topology, the ants were allowed to dig at full rate until the total length 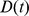 reached a value that was kept proportional to colony size 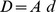, so that the tunnel length dug per ant was keeping the same value (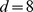 mm.ant^−1^). Typical examples of growth dynamics are shown in Movie S2 (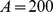), Movie S3 (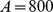), Movie S4 (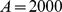) and Movie S5 (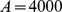).

**Figure 6:**
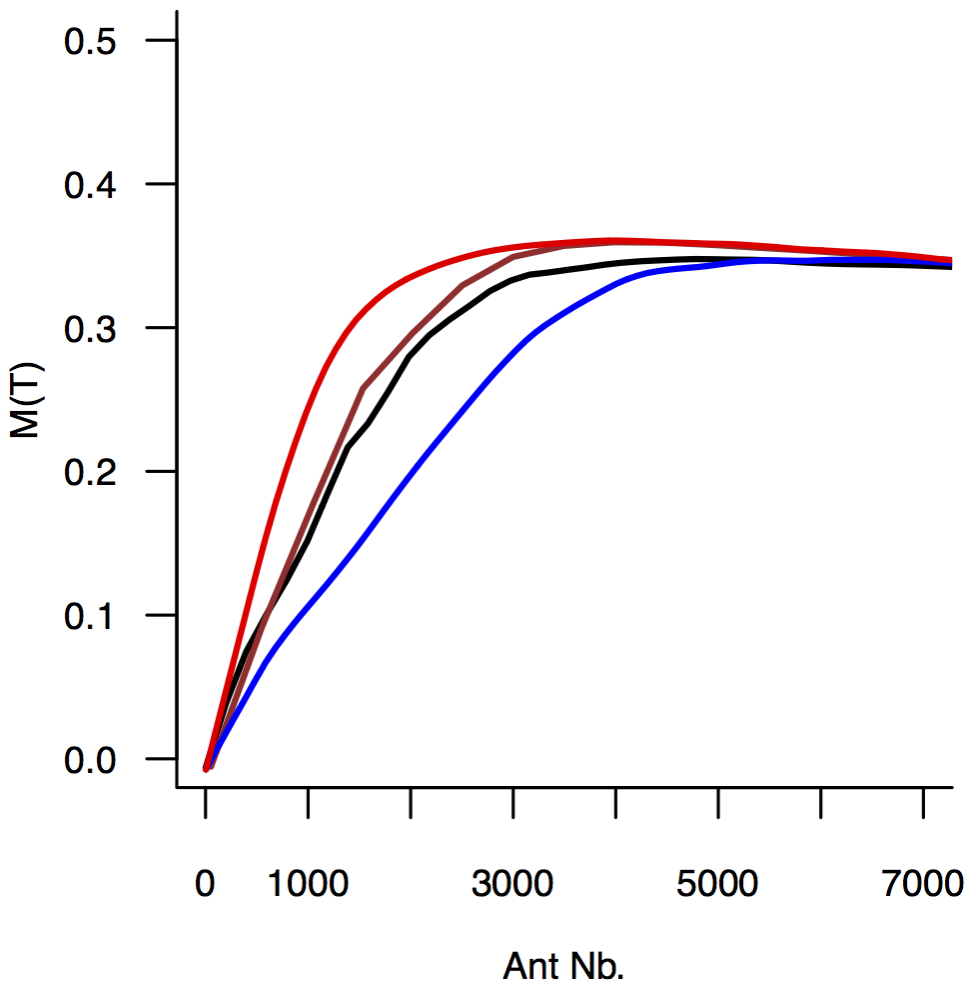
Meshedness 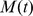 at the end of experiments as a function of the number of workers, predicted by the simplified model in a space with no boundary, either using the set of median values of parameters (brown line), picking randomly a parameter set among the experimental ones (black line), or using the set of median values but with the highest lateral nodes formation rate value (red line) or the lowest one (blue line). The number of workers was repeatedly picked uniformly between A = 1 and A = 7,000 for each condition and the lines report the corresponding tendency obtained by a lowess procedure (see Fig. S5). The meshedness increases as the number of workers increases, from tree-like structures (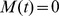) towards triangular networks (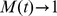) up to a saturation value. The predictions including the experimental variance of parameter estimations (black lines) show that the transition of 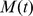 induced by the increase of 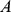 is robust to behavioural noise.

When there is no boundary, we also found that the meshedness increases with the number of workers and then reaches a plateau (fig. 6). The sensitivity to colony size may vary with the creation rate of lateral nodes 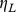 because a higher rate promotes more branching and as a consequence the meshedness becomes higher (fig. S5). However, these results are robust against noise in 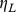. Since the meshedness is a structural property that does not depend on the length of the network or the number of nodes, in any case it will saturate to a value below 1. The plateau which is actually reached when the number of ants become very large (∼N>2000 in the present case) results from a balance between the spreading of the network area and the creation of new lateral tunnels inside the network. In large colonies, the meshedness is dominated by the geometrical structure of the network, i.e. by the distribution of triangles, squares, pentagons, etc. resulting from the tessellation of the plane. This distribution is likely to be affected by the dispersion in the orientation of new tunnels (a larger value of 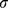 will promote a larger proportion of triangles); as a consequence the plateau value for the largest group sizes appears to be independent of 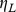.

## Discussion

In the experiments, the network structure results from a balance between (a) a positive feed-back: the longer the network, the higher the probability that ants start digging a new tunnel, and (b) a negative feed-back: as a consequence of spatial constraints, the creation of new tunnels on the periphery of the sand disk decreases as the number of existing peripheral tunnels increases. Both feedbacks are constrained by a diminishing workforce due to the decrease in ants' activity with time, which eventually stabilizes the network structure. The model predictions show a good agreement with the structural properties of networks at the end of experiments. In the model, the rates at which ants stop digging activity and start to dig new peripheral and internal tunnels remain constant with time. The collective digging process and the resulting tunnel network properties can thus be explained in terms of individual behaviours that are basically Markovian. This property has already been found in social insects in several collective behaviours, such as corpse aggregation in ants [27], division of labour and nest building in wasps [28], and aggregation in cockroaches [29], [30]. Note however that the *actual* probabilities of observing events during a given time interval, e.g. the emergence of a new lateral node, are constantly modulated by the evolution of the structure itself (i.e. the length of the network): even if the expression of individuals' behaviour is governed by constant rates, ants interact nonetheless with the evolving network structure, which in turn results from their digging behaviour. Hence collective digging belongs to the general class of stigmergic processes [31]. For instance, the inactivation of ants digging is likely to be triggered by the decreasing lack of nest space, not absolutely by time. Hence, the inactivation rate may simply vary among experiments because the networks grow at different speeds. As a consequence of the Markovian property, the result does not depend on the absolute temporal organization of the work, but rather on the sequence of the behavioural decisions over time. This would induce successive enlargements of nest size through bursts of activity, i.e. according to need (e.g. nest moving, [7]), or when new workers become available for digging. In this work we didn't investigate the mechanisms that drive the recruitment and the inactivation of active workers. This would have required a different experimental setup. The collective recruitment dynamics has been extensively studied in ants [32], and it may also be at work in nest digging. On the other hand, the inactivation process is likely to be triggered by the increase in empty space, which favours the aggregation of the ants within the new chambers [11].

As for the initiation of new galleries, either from the periphery or from the inside, we have used a kind of mean-field approach by considering that the digging sites were homogeneously distributed along the walls of existing tunnels at any time. This assumption proved to be highly relevant in view of the linear fits shown in fig. S2 and S3: on average new digging sites may appear everywhere with a probability that depends linearly with the length of walls. However one might question whether this statistical effect should be understood at the individual level or at the collective level. At the individual level this would imply that each ant decides on its own to start digging a new gallery. This is likely to be the case in our experimental setup since the sand was homogeneous, no particular location was distinct from any other one and the density of ants was kept low. At the collective level, fluctuations in the spatial distribution of workers may occur, such as transient traffic jams in the tunnels or the formation of workers aggregates and they could promote digging activity at some particular sites. However, such transient events are compatible with the mean-field approach that we have adopted provided that they are randomly distributed in space and that their temporal fluctuations occur on a shorter timescale in comparison to the growth rate of the network. Overall, this would yield the same growth dynamics, provided there is a limited feed-back effect of the network structure on the spatial distribution of workers, e.g. by affecting their movements.

Indeed, ant density can have a strong impact on digging. Toffin et al. have monitored the digging activity in the ant *Lasius niger* using a setup with a central digging hole [33]. They observed that ants first excavated a chamber with a circular shape. Digging tunnels under the chamber wall occurred later, once the chamber has reached a critical volume. The explanation was that a large number of digging ants relative to the nest area would promote a uniform digging activity because of the high density of ants along the initially limited surface of the nest. Later, as the nest area increases, the average density of ants falls down to a critical value where a transition may occur, and localized excavated buds appear because of amplification processes. To sum up, high worker density promotes a uniform digging activity while low worker density promotes localized digging and the formation of new tunnels. In our experiments, ants had initially access to a long wall (the periphery of the sand disk) and there was no crowding. As network grows over time, the density of ants gets even weaker because more space is made available. Indeed the linear fitting shown in figures S2 and S3 is an indication that there was no crowding effect in the beginning of experiments that would have prevented the formation of new galleries. This is further supported by observing that the behavioural parameters estimated from the dynamics in groups of A = 100 ants are similar to those found in groups of A = 200 ants (fig. S6).

It is expected that a non-homogeneous environment would affect the network topology of subterranean nests in natural conditions because a large number of physical factors can create templates, such as wind speed, temperature, humidity and carbon dioxide gradients. For instance, Bristow et al. [34] have established the existence of a linear stratification of temperature in the nests of the North America ants *Formica exsectoides*, from 25–30°C inside the mounds (30 cm above ground, in July) down to 10°C one meter deep into the ground. As activity levels are strongly influenced by temperature in ants, a three-dimensional extension of our model should integrate such factors. Moreover, Bollazzi et al. [35] showed that in the ant *Acromyrmex lundi*, the workers have a temperature-dependent digging behavior. In [36] they further showed that in *Acromyrmex ambiguous*, the workers plug or open tunnels as a function of the humidity level at the nest entrance. Subsequently, they showed that the regulation of the number of openings in the thatch covering the nest of *Acromyrmex heyeri* [37] was controlled by both temperature and humidity. All these templates may affect the decision of digging new galleries in natural environments, but they also should combine their effects with substrate heterogeneities, which are likely to be predominant in natural soils. In a recent study, Minter et al. [38] applied computer micro-tomography to monitor the digging behaviour in the ant *Lasius flavus*. When the substrate was composed of a single homogeneous layer, ants initiated branching from the first artificial vertical shaft, with two to four tunnels slanting downwards. Interestingly, when the material was deposited as several layers on top of each other, the ants preferentially build more horizontal tunnels at the inter-layers planes level, possibly seeking the path of least resistance. But even in homogeneous conditions, the very nature of the substrate can affect growth dynamics and hence the resulting shape of the nest, as it has been shown by Toffin et al. [39]. They found that shape transition from a round chamber to a ramified tunnels system depended on the cohesiveness of the material; in particular, a highly cohesive substrate was favouring the formation of branching structures.

The model that we have presented here can be considered as a reference model in homogeneous substrate conditions and in absence of crowding; thus it could be used to detect the effects due to the heterogeneity of material properties and/or the presence of templates in the environment. When substrate heterogeneities trigger the digging activity of ants, the new galleries must still be elongated, and it might be tricky to disentangle the causes of initiation from the causes of further elongation, especially when stochastic effects are prevalent. For instance, in the experiments performed with stacked substrate layers described in [38], tunnels tend to be dug at the interfaces between layers. When there were five layers, they found on average four peaks of probability of finding a gallery at the four corresponding interfaces. However, in each particular network (Fig. S3 in [38]) only one or two tunnels had been stochastically initiated and then elongated among the four possible ones. It is then difficult to assess whether the heterogeneities between layers enhanced the initiation of new tunnels or their growth rate. A modified version of our model designed to suit new experimental conditions (3D vs. 2D environment, ant species, colony size) would be a useful tool to disentangle such template effects. We point out that 3D extensions of our model should also pay attention to the special role played by gravity since it can affect the spatial distribution of workers and its dynamics by promoting a vertical motion of ants [40]. If a temperature gradient exists in the environment, it may also induce a stratification of digging activity, with a higher working rate and/or lower inactivation rate in the top parts of the nest where the temperature is higher than in the bottom parts. This could in turn lead to a higher meshedness in the top parts and “top-heavy” patterns reported in several species (Table 1).

Our experiments showed that the meshedness of the tunnelling networks increases with the number of workers in the colony. The model was able to reproduce quite well the structural changes in the network properties, even when the geometrical details describing the growth of nodes were discarded, and when the digging started from a single starting point. The model thus confirms that meshedness in tunnelling networks increases as a consequence of an increase in colony size. A high meshedness shortens the inter-nodes distance since it increases the number of shortcuts between distant nodes. Hence, there is no doubt that bigger nests should promote a higher meshedness to maintain functional supply systems such as food and water transportation [41]. The present study suggests that the stigmergic dynamics can automatically generate this structural change when the population of workers increases, with no need to change individuals' behaviours to get these properties. Obviously, introducing an additional modulation of individual behaviour, such as a greater ability to dig as a function of age [20], could further regulate the nest morphology. However, since it is a robust feature resulting from the dynamics of the observed process, the principle of a colony-size-dependent meshedness should hold not only for the growth of one particular colony, but also across the species-related diversity in mature colony sizes. Hence, as for the division of labour in ant colonies [42], [43], the colony size should be considered as a key factor explaining the topological properties of subterranean ant nests.

## Supporting Information

Data S1 The graphs data are reported in the zip file S1.zip. Two directories are given: the directory “Final” contains the final state of the graph for all experiments, with file labels in the form “A050-01” for the group size 50, replicate 1; the directory “TimeSequences” contains the time series of networks for the group sizes 100 and 200 with one directory per experiment (same labelling convention). Within each directory, the series of 217 snapshots are given in the order. Each graph is reported in a “.gra” file which contains: the list of nodes (vertices) describing for each node: “noeud”, NodeIdent, NodeType, NodeX, NodeY, NodeRadius, followed by the list of tunnels between nodes (edges) describing for each edge: “arc”, Node1Ident, Node2Ident, Width. The Node1Ident and Node2Ident correspond to the labels NodeIdent in the list of nodes.
(ZIP)

Figure S1Time evolution of 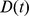 (solid line) and the corresponding prediction of the model (dashed line). y-axis: 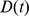 in mm, x-axis: time in minutes.(TIFF)

Figure S2Number of peripheral nodes 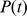 as a function of 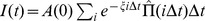. Lines indicate the regression whose slope is 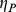.(TIFF)

Figure S3Number of lateral nodes 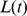 as a function of 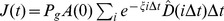. Lines indicate the regression whose slope is 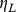.(TIFF)

Figure S4A: time evolution of network length are reported for each experiments with A = 200 (orange lines). The average evolution is reported in black. The model's predictions using the average parameters is reported in blue. The brown line reports model's predictions with 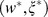 adjusted to reflect the average time course. B: The meshedness of the observed networks is reported as a function of their length for all group sizes (green: A = 50, red: A = 100, blue: A = 200). The deep blue line indicates the corresponding linear regression. Orange line: linear regression for the simulations using the complete collection of parameters sets. Orange squares: corresponding means for each group size. Light blue line and squares: same quantities for the simulations using the set of average parameters, in particular mean activity parameters (blue line on left panel). Brown line and squares: same quantities for the simulations using average branching rates, and activity parameters fitted to reflect the average activity (brown line on left panel). Gray polygons: confidence ellipses containing respectively 99% (dark gray), 95% (medium gray) and 50% (light gray) of the simulated points closest to the regression line of the latter case.(TIFF)

Figure S5Meshedness at the end of experiments 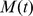 as a function of the number of workers, predicted by the simplified model in a space with no boundary, either using (A) the set of median values of parameters, (B) picking randomly a parameter set among the experimental ones, (C) using the set of median values but with the highest lateral nodes formation rate value or (D) the lowest one. The number of workers was repeatedly picked uniformly between A = 1 and A = 7,000 for each condition (one dot per simulation) and the corresponding tendency was obtained by a lowess procedure (red lines).(TIFF)

Figure S6 To check for an effect of the density of ants on their behaviour, we estimated the behavioural parameters from the dynamics observed in experiments with A = 100 (red dots) and we compared them to the estimation of the same parameters found for A = 200 (blue dots). Both sets appear consistent.
(TIFF)

Movie S1Typical dynamics of a network dug by ants (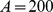, real duration 3 days).(MOV)

Movie S2 Typical evolution of the network dug by 200 ants in a space with no boundary.
(MOV)

Movie S3 Typical evolution of the network dug by 800 ants in a space with no boundary.
(MOV)

Movie S4 Typical evolution of the network dug by 2000 ants in a space with no boundary.
(MOV)

Movie S5 Typical evolution of the network dug by 4000 ants in a space with no boundary.
(MOV)
