# Supplementary figures and images for: The Role of Colony Size on Tunnel Branching Morphogenesis in Ant Nests
Source: PLoS One. 2014 Oct 15;9(10):e109436. doi: 10.1371/journal.pone.0109436 (PMC4198125; doi:10.1371/journal.pone.0109436)

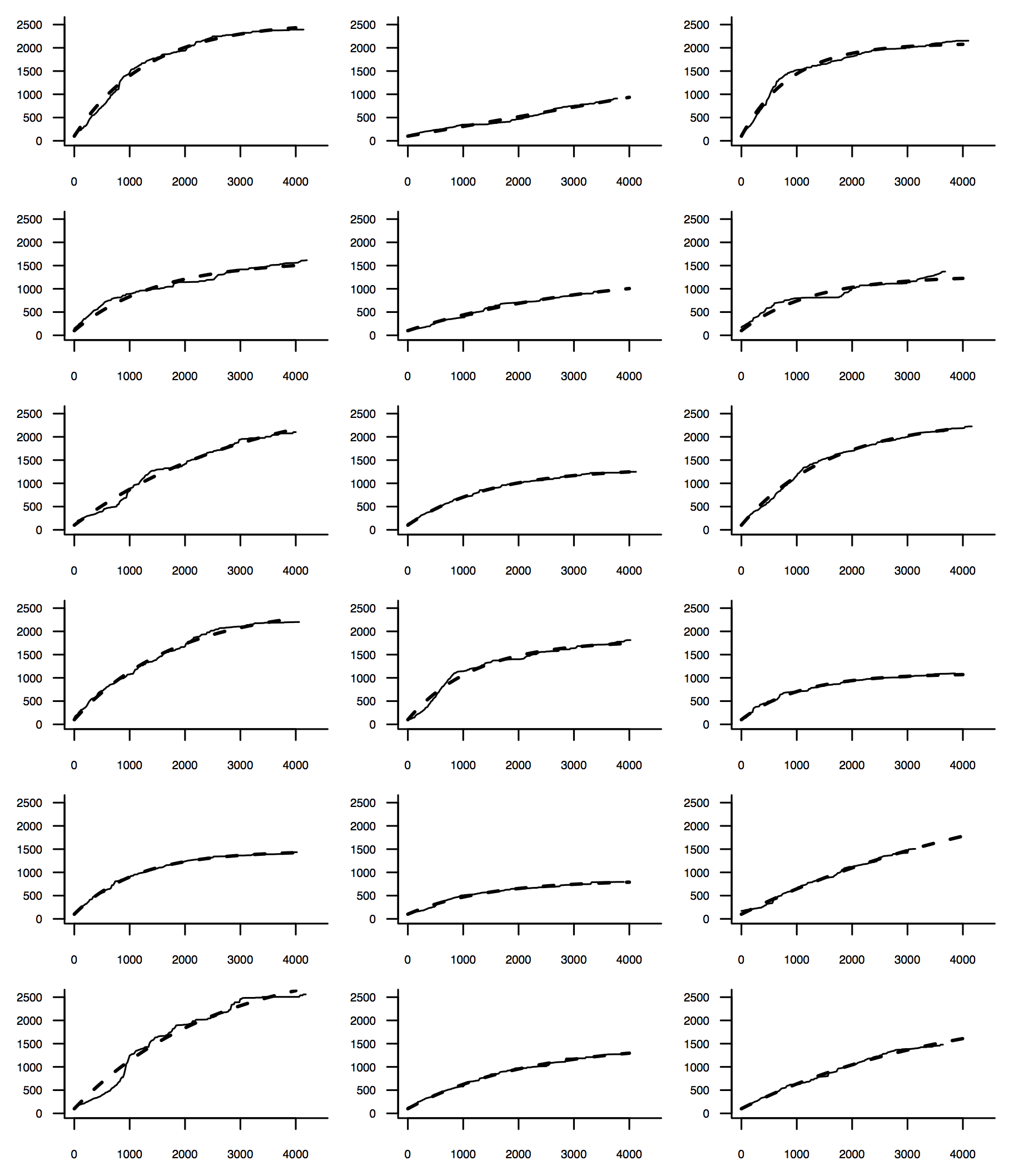

Supplement: Figure S1 — Time evolution of (solid line) and the corresponding prediction of the model (dashed line). y-axis: in mm, x-axis: time in minutes. (TIFF) [file pone.0109436.s002.tiff]

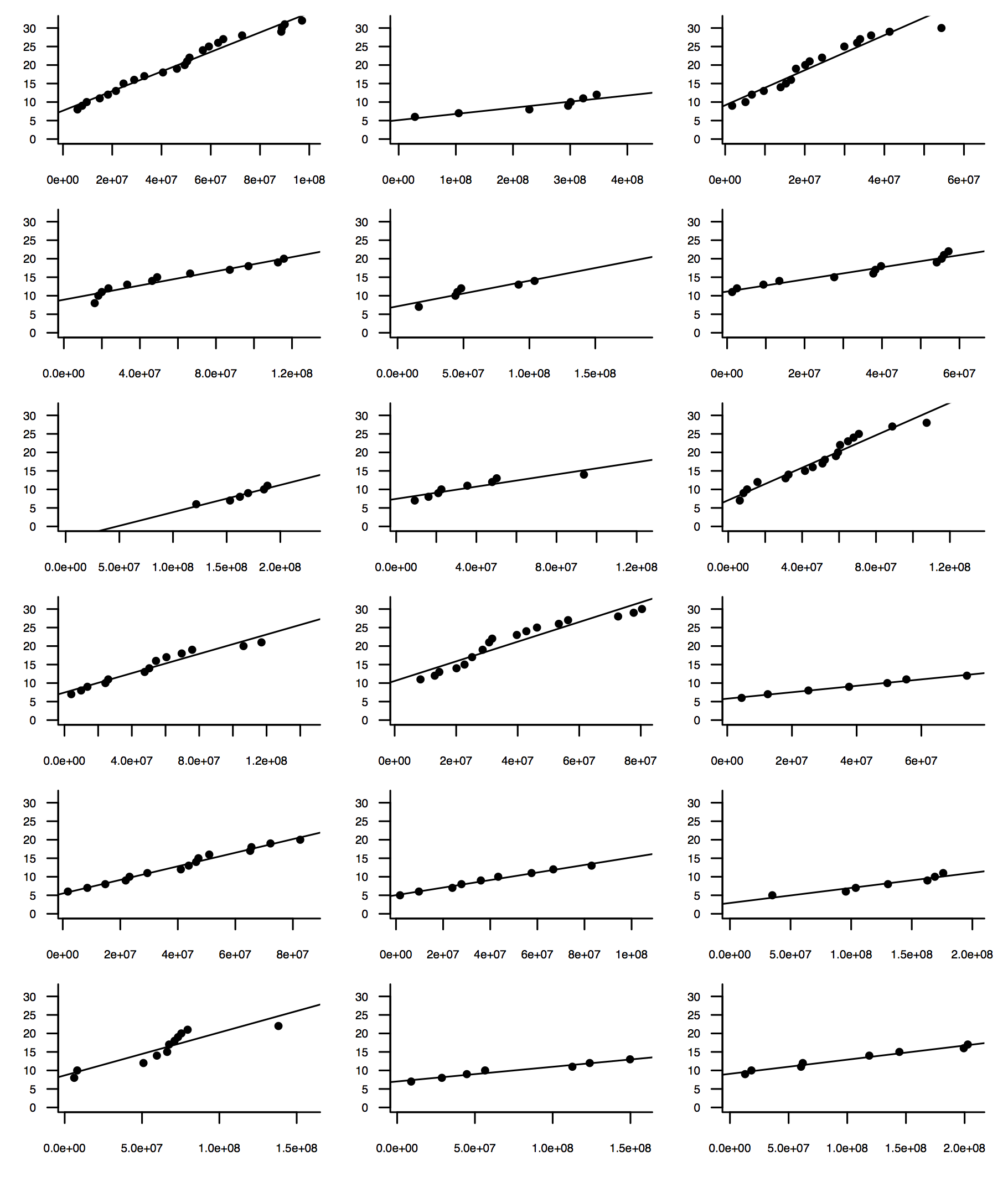

Supplement: Figure S2 — Number of peripheral nodes as a function of . Lines indicate the regression whose slope is . (TIFF) [file pone.0109436.s003.tiff]

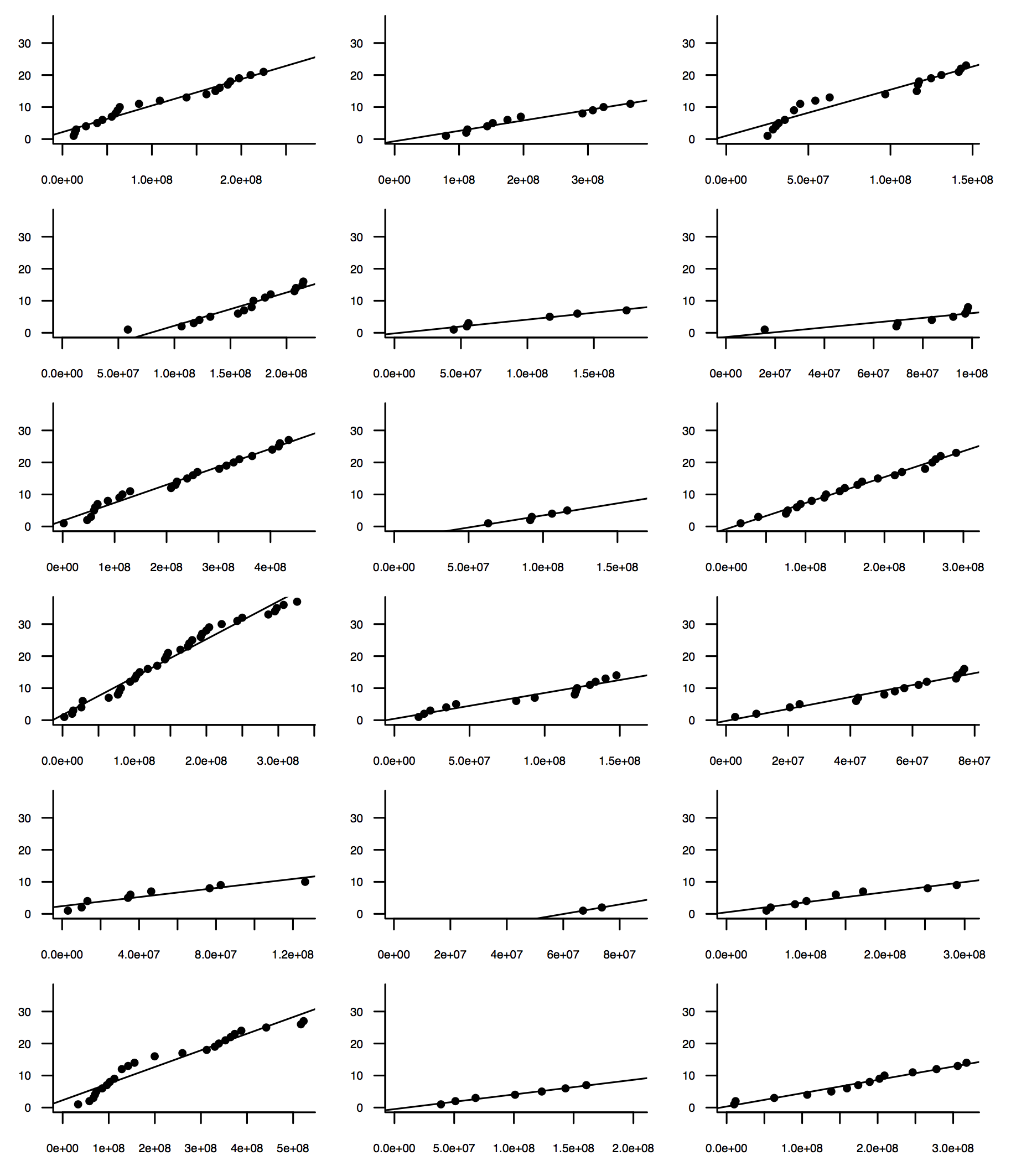

Supplement: Figure S3 — Number of lateral nodes as a function of . Lines indicate the regression whose slope is . (TIFF) [file pone.0109436.s004.tiff]

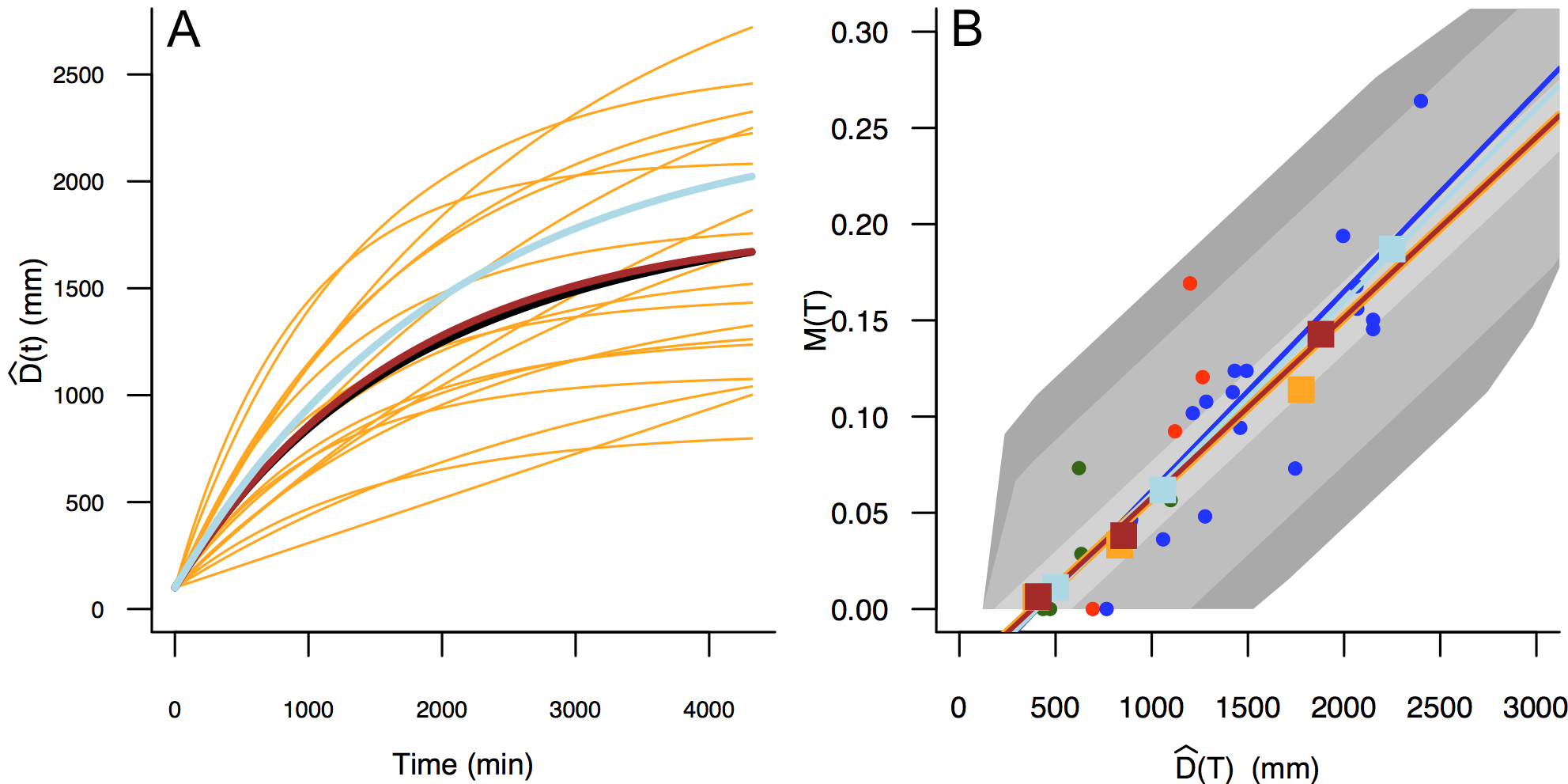

Supplement: Figure S4 — A: time evolution of network length are reported for each experiments with A = 200 (orange lines). The average evolution is reported in black. The model's predictions using the average parameters is reported in blue. The brown line reports model's predictions with adjusted to reflect the average time course. B: The meshedness of the observed networks is reported as a function of their length for all group sizes (green: A = 50, red: A = 100, blue: A = 200). The deep blue line indicates the corresponding linear regression. Orange line: linear regression for the simulations using the complete collection of parameters sets. Orange squares: corresponding means for each group size. Light blue line and squares: same quantities for the simulations using the set of average parameters, in particular mean activity parameters (blue line on left panel). Brown line and squares: same quantities for the simulations using average branching rates, and activity parameters fitted to reflect the average activity (brown line on left panel). Gray polygons: confidence ellipses containing respectively 99% (dark gray), 95% (medium gray) and 50% (light gray) of the simulated points closest to the regression line of the latter case. (TIFF) [file pone.0109436.s005.tiff]

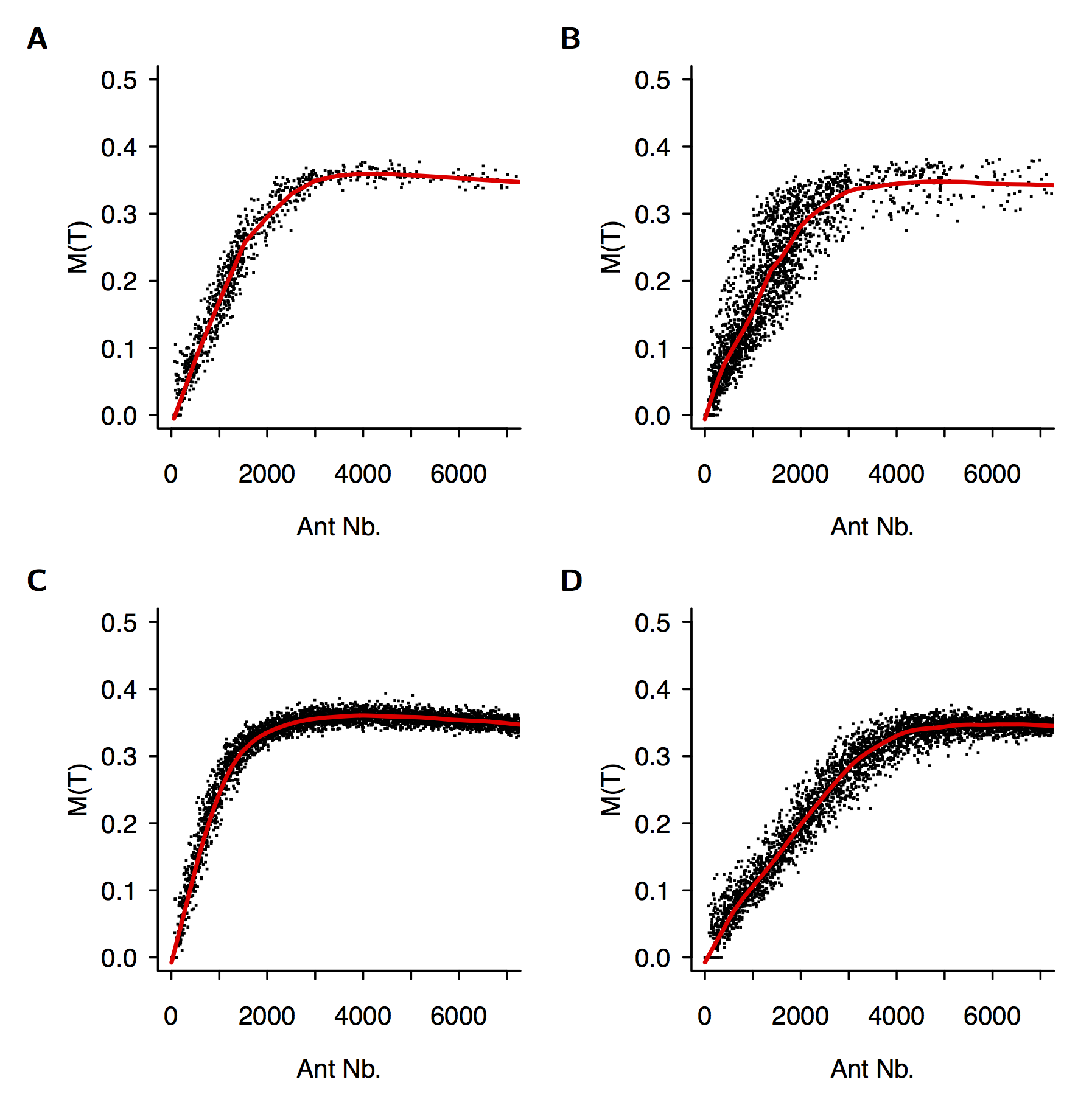

Supplement: Figure S5 — Meshedness at the end of experiments as a function of the number of workers, predicted by the simplified model in a space with no boundary, either using (A) the set of median values of parameters, (B) picking randomly a parameter set among the experimental ones, (C) using the set of median values but with the highest lateral nodes formation rate value or (D) the lowest one. The number of workers was repeatedly picked uniformly between A = 1 and A = 7,000 for each condition (one dot per simulation) and the corresponding tendency was obtained by a lowess procedure (red lines). (TIFF) [file pone.0109436.s006.tiff]

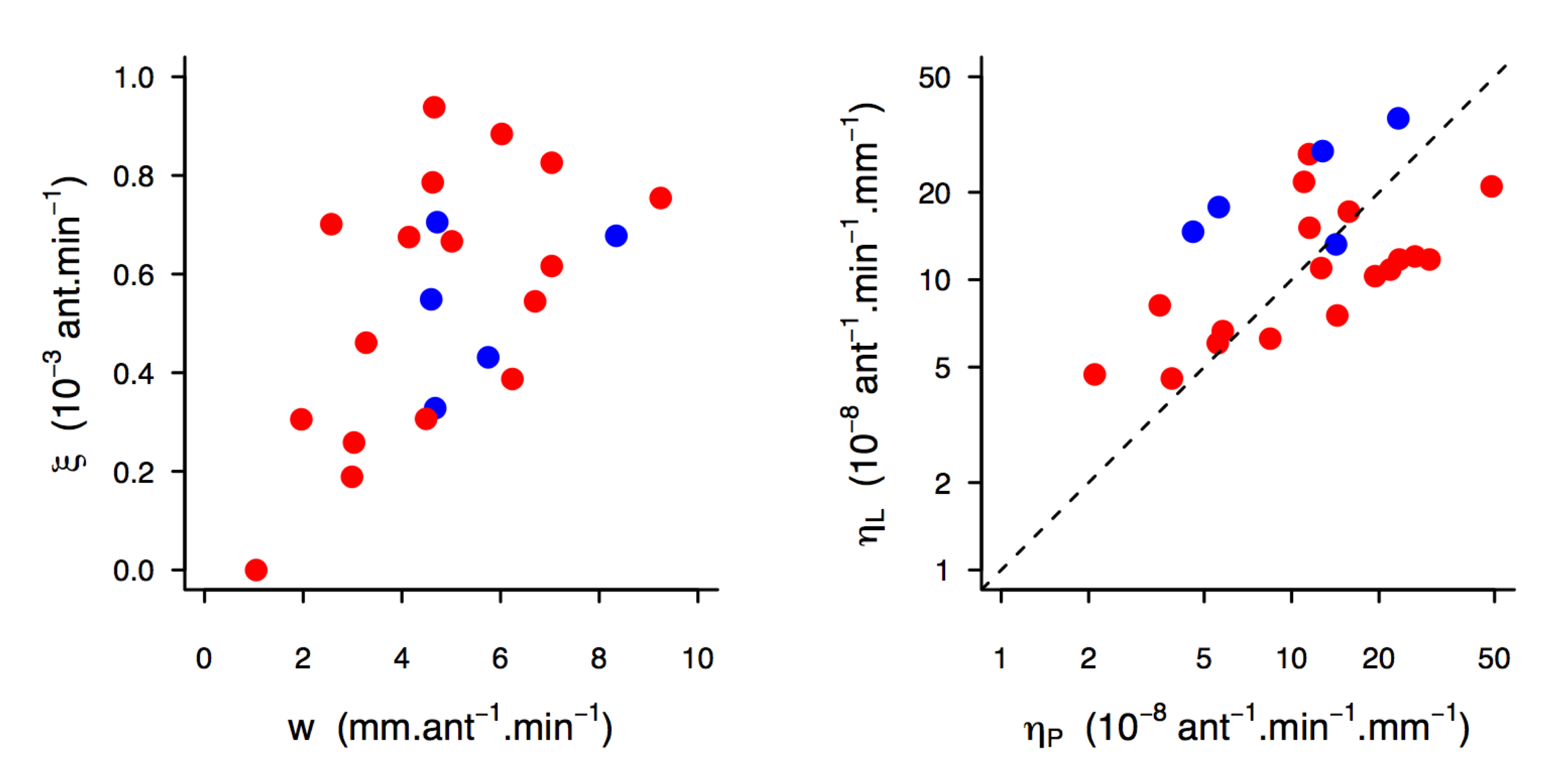

Supplement: Figure S6 — To check for an effect of the density of ants on their behaviour, we estimated the behavioural parameters from the dynamics observed in experiments with A = 100 (red dots) and we compared them to the estimation of the same parameters found for A = 200 (blue dots). Both sets appear consistent. (TIFF) [file pone.0109436.s007.tiff]
